# Development of Pancreatic Ductal Adenocarcinoma Associated with Intraductal Papillary Mucinous Neoplasia

**DOI:** 10.5402/2011/940378

**Published:** 2011-12-08

**Authors:** Kazuo Inui, Junji Yoshino, Hironao Miyoshi, Takashi Kobayashi, Satoshi Yamamoto

**Affiliations:** Department of Internal Medicine, Second Teaching Hospital, Fujita Health University School of Medicine, 3-6-10, Otobashi, Nakagawa-ku, Nagoya 454-8509, Japan

## Abstract

We retrospectively investigated the incidence of pancreatic ductal adenocarcinoma among patients with intraductal papillary mucinous neoplasms of the pancreas. Based on imaging in 195 such patients, we chose surgery as initial treatment for 54, and periodic evaluation over 6 to 192 months (mean, 52) for 141. In 6 of the 141 patients observed for intraductal papillary mucinous neoplasm (4.2%), pancreatic ductal adenocarcinoma developed. Further, careful monitoring for cancer occurrence in the remnant pancreas proved essential in the surgical resection group; 2 of 26 patients (7.7%) subsequently developed pancreatic ductal adenocarcinoma in the remnant pancreas, at 41 months and 137 months after surgery. Serial observation of patients with intraductal papillary mucinous neoplasms by contrast-enhanced computed tomography or magnetic resonance cholangiopancreatography therefore is critical, whether or not surgical treatment initially was performed.

## 1. Introduction

Asymptomatic patients with intraductal papillary mucinous neoplasms (IPMN) of the pancreas are common in Japan, as these tumors are detected by widely conducted ultrasonographic mass surveys. Since IPMNs include several pathologic types including invasive carcinoma, carcinoma in situ, adenoma, and hyperplasia, one needs to distinguish benign from malignant lesions to avoid unnecessary surgery. On the other hand, in 2002, Yamaguchi et al. [1] reported an important relationship between IPMN and ductal adenocarcinoma of the pancreas: IPMN is a strong risk factor for pancreatic cancer. Our group [2] has suggested that serial observations should be performed at every 6-month intervals in patients with IPMN of the branch duct type. We presently investigated usefulness of intraductal ultrasonography (IDUS) as a precise diagnostic modality for IPMN and also examined long-term incidence of pancreatic ductal adenocarcinoma (PDAC) associated with IPMN of the branch duct type.

## 2. Patients and Methods

We treated 208 patients with PDAC from 1990 to 2009 at our hospital. The treatment was surgical in 64 patients. We analyzed them in terms of age, gender, tumor staging, resection rate, and association with IPMN.

**Figure 1:**
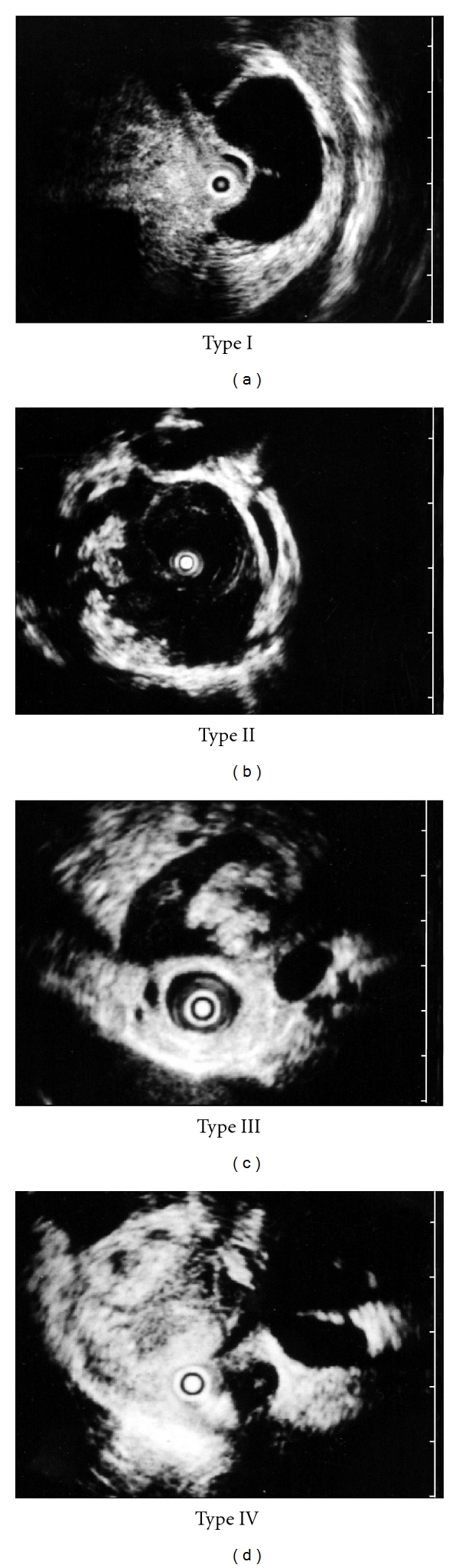
Classification of IPMN of the branch duct type into 1 to 4 types based upon images obtained by EUS and/or IDUS: type I, with no mural nodule; type II, with a mural nodule-elevated lesion less than 5 mm; type III, with a mural nodule elevated at least 5 mm; or type IV, solid tumor with a mixture of high and low echogenicity in the pancreatic parenchyma.

We also treated 195 patients with IPMN at our hospital over the same period. We diagnostically evaluated IPMN using ultrasonography, endoscopic ultrasonography (EUS), intraductal ultrasonography (IDUS), endoscopic retrograde cholangiopancreatography (ERCP), contrast-enhanced computed tomography (CECT), and magnetic resonance cholangiopancreatography (MRCP). We used mainly EUS and IDUS in deciding about surgical treatment in patients with IPMN [3]. We performed IDUS using a miniature ultrasonic probe with frequency of 20 MHz, developed by Olympus Medical Systems (Tokyo, Japan). We classified 4 images (Figure 1) obtained by EUS and/or IDUS as type I, with no mural nodule; type II, with a mural nodule elevated less than 5 mm; type III, with a mural nodule at least 5 mm; or type IV, solid tumor with a mixture of high and low echogenicity in the pancreatic parenchyma [4]. Type III suggested intraductal papillary mucinous adenoma (IPMA), while type IV suggested intraductal papillary mucinous adenocarcinoma (IPMC). We recommended surgical treatment for patients with type III or IV images, and follow-up examination at 6-month intervals for patients with type I or II.

According to the indications outlined above, we performed surgical treatment for 54 patients, and periodic evaluations including ultrasonography, CECT, and/or MRCP, over 6 to 192 months (mean, 52) for 141 patients. We analyzed patients with IPMN in terms of age, gender, pathologic findings, and incidence of PDAC.

## 3. Results

### 3.1. Pancreatic Ductal Adenocarcinoma

Sixty-four patients with PDAC underwent surgical treatment; the resection rate was only 30.1%. Men accounted for 42 patients and women for 22. Ages ranged from 33 to 80 years (mean, 52). In 144 patients with PDAC, surgical treatment was not performed because of advanced tumor stage. Eighty-eight of these patients were men and 60 were women; ages ranged from 47 to 94 years (mean, 71). Patients undergoing resection were younger than the others (*P* < 0.001). Only 11 tumors were considered small (less than 2 cm in diameter), including 4 T1 cases (limited to the pancreas). According to the TNM classification, 6 surgery patients were at stage I; 6, stage II, 18, stage III; and 34, stage IV. Of these 64 surgical patients with PDAC, 4 (6.3%) had associated IPMN; all 4 underwent resection (2, stage I; 1, stage III; and 1, stage IV).

### 3.2. Case 1: A Patient with PDAC and IPMN of the Branch Duct Type

**Figure 2:**
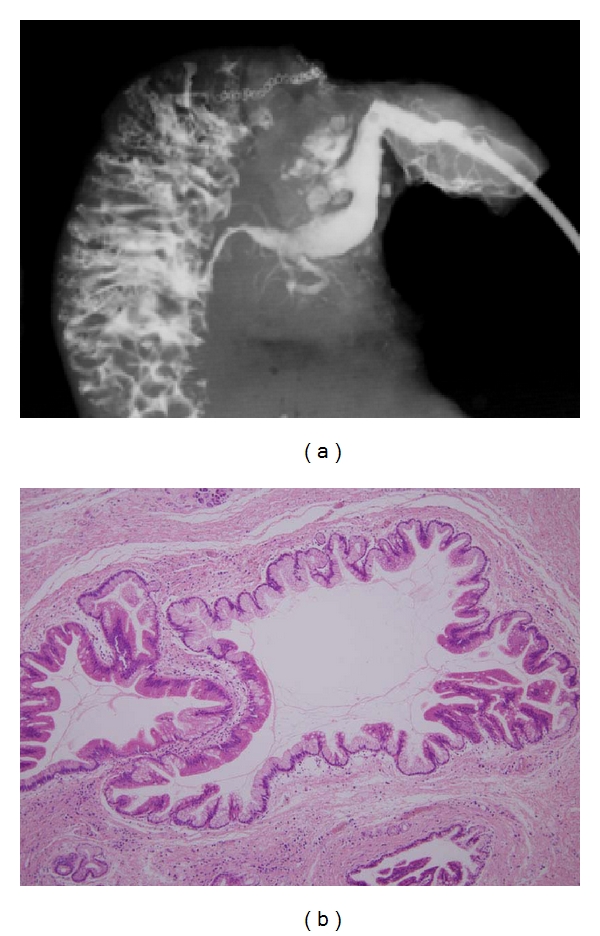
Case 1: (a) Pancreatogram showing dilation of the main pancreatic duct and cystic dilation of the branch duct. (b) Pathologic examination disclosed IPMA. Haematoxylin and eosin high magnification.

**Figure 3:**
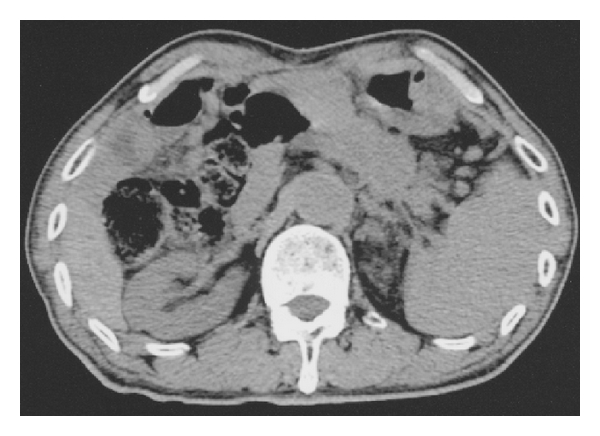
Case 1: A noncontrast CT 137 months after surgical treatment demonstrating enlargement of the remnant pancreas.

**Figure 4:**
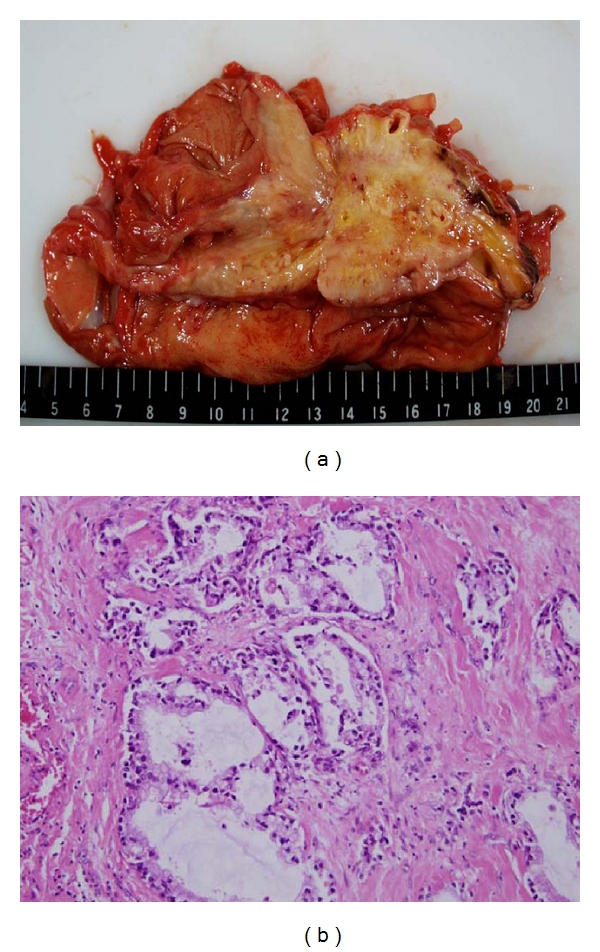
(a) Macroscopic findings at autopsy. (b) Pathologic examination disclosed tubular adenocarcinoma of the pancreas. Haematoxylin and eosin, high magnification.

In a 55-year-old man, ultrasonographic mass screening detected a multilocular cystic lesion in the head of the pancreas; ERCP revealed dilation of the main pancreatic duct and dilation of the branch duct. Pancreatoduodenectomy was performed; pathologic examination disclosed IPMA (Figure 2). Ultrasonography and noncontrast CT 137 months after surgical treatment detected enlargement of the remnant pancreas (Figure 3). The patient declined additional resection and died 11 months later. Pathologic examination at autopsy disclosed tubular adenocarcinoma of the pancreas (Figure 4).

### 3.3. Intraductal Papillary Mucinous Neoplasms of the Pancreas

Surgery was performed in 54 patients with IPMN. Twenty-nine patients were men and 25 were women. Age ranged from 26 to 79 years (mean, 62). Pathologic examinations identified hyperplasia in 5 patients; adenoma in 28, including 6 in whom it was associated with PDAC; and adenocarcinoma in 21 (10 carcinomas in situ and 11 invasive carcinomas). Of the 11 PDAC considered small, 3 were detected in patients with IPMN. Among 28 patients with IPMA who underwent surgical treatment, 18 were men and 10 were women; ages ranged from 26 to 79 years (mean, 64). In 2 of 28 patients with IPMA (7.1%), pathologic examinations demonstrated PDAC at a site other than that of IPMA. In another 2 of 26 patients with IPMA but no synchronous PDAC (7.7%), PDAC subsequently developed in the remnant pancreas, at 41 and 137 months after surgery.

Among 141 patients with IPMN who underwent periodic evaluation over 6 to 192 months (mean, 52), 61 were men and 80 were women. Ages ranged from 35 to 88 years (mean, 61). In 6 of 141 patients with IPMN (4.2%), PDAC developed during serial observation periods (mean duration of observation, 6 to 103 months (mean, 47). Three of these patients underwent surgical treatment; the other 3 did not because of advanced tumor stage (2 patients in stage I, and 4 in stage IV).

### 3.4. Case 2: A Patient with IPMN Who Developed PDAC during Observation

**Figure 5:**
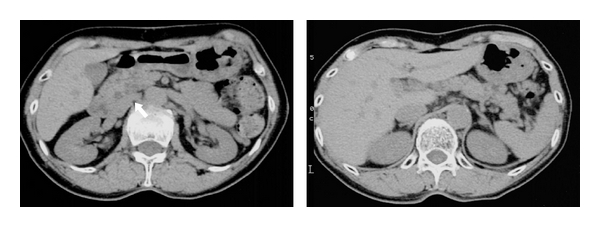
Case 2: Noncontrast CT showing a small multilocular cystic lesion in the head of the pancreas and no lesions in the tail of the pancreas.

**Figure 6:**
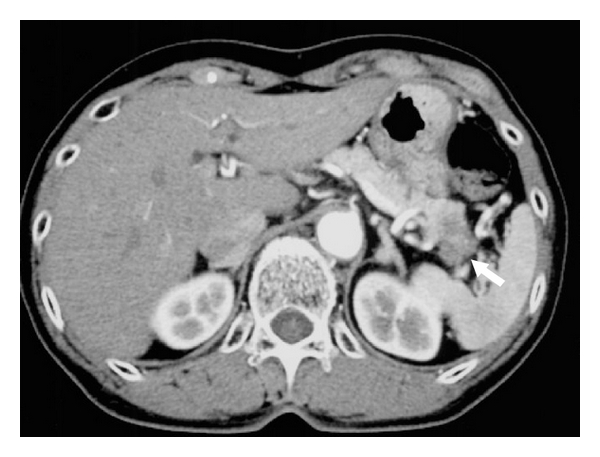
Case 2: Contrast CT 6 months after the first CT demonstrating a small tumor as a low-density area in the tail of the pancreas.

**Figure 7:**
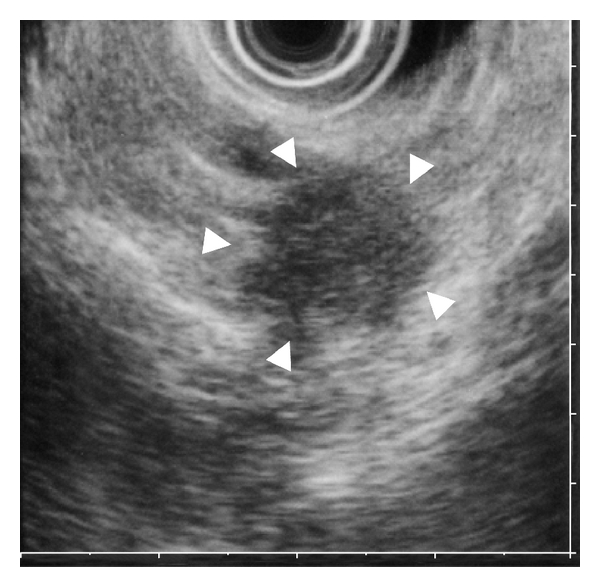
EUS disclosing a small hypoechoic mass, 20 mm in diameter, in the tail of the pancreas.

**Figure 8:**
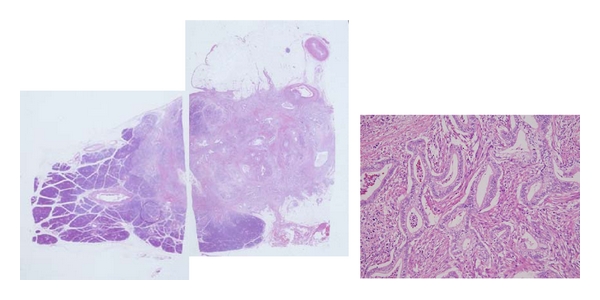
Pathologic findings diagnostic of well-differentiated tubular pancreatic adenocarcinoma, not extending beyond the pancreas. Haematoxylin and eosin: low and high magnification.

In a 79-year-old woman, a noncontrast CT detected a small multilocular cystic lesion in the head of the pancreas (Figure 5). ERCP showed dilation of the main pancreatic duct, but not cystic lesions, and EUS identified a multilocular cystic lesion in the head of the pancreas (but none in the tail). Results of cytologic examination of pancreatic juice were considered class 3. Contrast CT 6 months after the first CT detected a small tumor as a low-density area in the tail of the pancreas (Figure 6). EUS showed this as a hypoechoic mass, 20 mm in diameter, in the tail of the pancreas (Figure 7). The pathologic diagnosis was well-differentiated tubular pancreatic adenocarcinoma with no extension beyond the pancreas (stage I; Figure 8).

## 4. Discussion

IPMN first was described by Ohhashi et al. [5] in 1982 as a mucin-producing pancreatic tumor. Several other publications followed [6–11], and an international consensus statement was issued in 2006 [12]. Reported patients with IPMN usually were symptomatic, especially when the tumor was malignant [13]. In Japan, however, ultrasonographic mass screening for digestive cancers conducted nationwide; so patients with IPMN are detected in an asymptomatic condition. Appropriate therapeutic indications for asymptomatic patients diagnosed by mass screening are as important as correct diagnoses.

 Recent advances in diagnostic imaging have been helpful determining therapeutic procedures. CECT using multidetector-row-computed tomography (MDCT) can detect a mural nodule in a cyst, making it useful in differential diagnosis between benign and malignant lesions [14]. Development of MRCP promises reliable delineation of communications between the main pancreatic duct and cystic lesions. MRI allows more confident assessment of morphology of small cysts than MDCT, but accuracy of the two imaging techniques for characterization of most cysts is comparable [15]. EUS is reported to be a reliable method for differential diagnosis of IPMN [16–18]. Preoperative diagnosis of the malignant potential of IPMN is of growing importance because pancreatic surgery has its complications [19]. We reported usefulness of IDUS for differential diagnosis of IPMN between benign and malignant lesions [2]. In unpublished study, we performed IDUS for 17 patients with IPMN treated by surgical resection; elevations of lesions measured by IDUS were 3.8 ± 1.3 mm for IPMA but were significantly greater for IPMC (8.3 ± 3.0 mm, *P* < 0.05). Corresponding elevations measured at pathologic examination were 2.6 ± 1.0 and 6.5 ± 2.8 mm, respectively (*P* < 0.01). Elevations of lesions determined by IDUS were less than 5 mm in 6 of 7 IPMA. We suggested that patients with a papillary nodule showing elevation of 5 mm more should be treated surgically. We usually used IDUS in determining the therapeutic approach for IPMN.

 Clinicians should note the possibility of coexisting or subsequently developing PDAC in patients with IPMA of the branch duct type. When Yamaguchi et al. [1] reported the relationship between IPMN and ductal adenocarcinoma of the pancreas, their 7 patients with both tumor types represented 9.2% of the 76 patients with IPMN and 9.1% of the 77 patients with PDAC. All 7 IPMNs were of the branch duct type with a mean diameter of 3.0 cm. In our series, of 141 serially observed patients with IPMN, 6 (4.2%) developed PDAC. Vigilance for cancer occurrence in the remnant pancreas after surgical resection is necessary. Among 26 of our patients, 2 (7.7%) subsequently developed PDAC in the remnant pancreas, after 41 and 137 months. Maguchi et al. [20] reported that PDAC developed in 7 of 349 patients with IPMN of the branch duct type (2%) during observation periods ranging from 1 to 16.3 years (median, 3.7). Sawai et al. [21] reported EUS followed-up for at least 2 years (median 59 months) in 103 patients with IPMN of the branch duct type; 6 patients (5.8* *%) developed pancreatic cancers during follow-up, specifically IPMC in 4 and PDAC in 2 patients. On the other hand, our recurrence rate following partial pancreatectomy for benign IPMN was similar to that reported by the Mayo Clinic: 5 of 60 patients (8%) with a median follow-up duration of 37 months [22]. Patients with IPMA treated by resection indeed require long-term follow-up.

When IPMN is detected, EUS is recommended for differential diagnosis. However, how and when should serial observations be made in patients with IPMN? Diagnostic precision of EUS depends upon the skill of individual endoscopists. On the other hand, MRCP and MDCT show high accuracy in classifying cysts into mucinous and nonmucinous categories and perform similarly in estimating histologic aggressiveness [15]. We therefore recommend performing MRCP or CECT with MDCT every 6 months. If changes in morphology of cystic lesions occur, EUS and/or IDUS should be performed again. Further studies should better define choice of modality and duration of serial observation.

Early detection of PDAC in patients without symptoms is the most important goal for improving prognosis of patients with pancreatic cancer. In addition to the ordinary risk factors including IPMN, individuals with a family history of PDAC and hereditary syndromes are expected to be entered into the screening protocol [23].

## 5. Conclusions

EUS and IDUS are recommended as a further evaluation for IPMN detected by mass screening. Because IPMN is an important risk factor for PDAC, clinicians should be aware of the possible coexistence or development of PDAC in patients with IPMN, especially the branch duct type. Serial observation of patients with IPMN by MDCT or MRCP is necessary whether or not initial surgical treatment is performed.
